# Neuropharmacology of Vestibular System Disorders

**DOI:** 10.2174/157015910790909511

**Published:** 2010-03

**Authors:** Enrique Soto, Rosario Vega

**Affiliations:** Institute of Physiology, Autonomous University of Puebla, México

**Keywords:** Inner ear, Vertigo, Dizziness, Ménière's disease, vestibular nuclei, hair cells, excitatory amino acids.

## Abstract

This work reviews the neuropharmacology of the vestibular system, with an emphasis on the mechanism of action of drugs used in the treatment of vestibular disorders. Otolaryngologists are confronted with a rapidly changing field in which advances in the knowledge of ionic channel function and synaptic transmission mechanisms have led to the development of new scientific models for the understanding of vestibular dysfunction and its management. In particular, there have been recent advances in our knowledge of the fundamental mechanisms of vestibular system function and drug mechanisms of action. In this work, drugs acting on vestibular system have been grouped into two main categories according to their primary mechanisms of action: those with effects on neurotransmitters and neuromodulator receptors and those that act on voltage-gated ion channels. Particular attention is given in this review to drugs that may provide additional insight into the pathophysiology of vestibular diseases. A critical review of the pharmacology and highlights of the major advances are discussed in each case.

## INTRODUCTION

Vertigo, dizziness and imbalance are the main symptoms of vestibular disorders. These symptoms can lead to physical consequences such as reduced postural control and falls and to psychological and psychiatric consequences such as depression, anxiety, panic, agoraphobia, and cognitive defects, especially in the elderly [98]. The goal of the treatment of vestibular disorders should be to control symptoms, reduce functional disability and improve the quality of life of the patients. This article reviews the neuropharmacology of vestibular disorders with an emphasis on the mechanism of action of drugs used in the treatment of such disorders and with emphasis on drugs that, due to their mechanisms of action, may provide additional insight into the physiopathology of vestibular disease. Drugs that by their systemic effect could secondarily affect vestibular function are not included in the review.

Therapeutic options for the treatment of vestibular diseases are restricted to pharmacological treatments, repositioning maneuvers, surgical particle liberation, and rehabilitation [96, 164]. At peripheral end organs, drugs that act on the vestibular apparatus have diverse cellular targets. These targets are involved in the homeostasis of liquids and electrolytes in the inner ear, in the regulation of blood flow and cellular homeostasis and survival, and in the modification of sensory processes including mechanoelectrical transduction in hair cells and postransductional processing of sensory information. In vestibular nuclei, in the central nervous system, drug effects are more difficult to define due to the complexity of the neural systems involved in vestibular information processing. We have centered our analysis of drugs with central effects on those acting on neurotransmitter receptors and ion channels.

From a pharmacological point of view, it is important to emphasize that the central portion of the vestibular system is isolated from the systemic blood flow by the blood-brain barrier, whereas the periphery of the vestibule is isolated by the blood-labyrinthic barrier. Because of this, some drugs can affect one region without affecting the other. Strategies have also been designed that allow the local administration of some drugs, thus limiting their systemic effects.

## DRUGS WITH EFFECTS ON NEUROTRANSMITTERS AND NEUROMODULATOR RECEPTORS

**Fig. (1):**
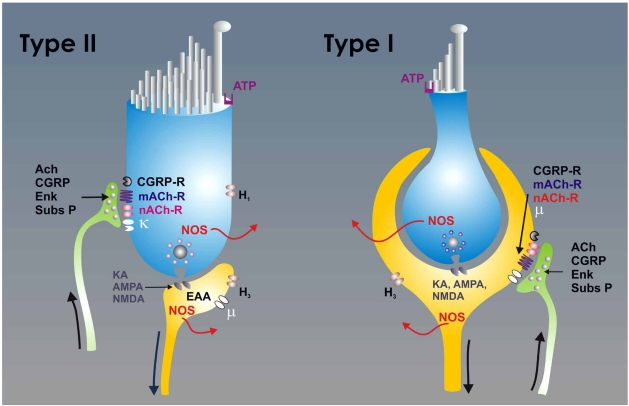
The scheme depicts the synaptic relationships of type I (right) and type II (left) hair cells. The type I hair cells are characterized by the large chaliceal afferent innervation that covers its basolateral surface. The efferent fibers make synaptic contacts with the external surface of the calyx in the afferent neuron. In type II hair cells, the afferent neurons form button type synapses, and the efferent neurons make synaptic contact directly upon the hair cell body. The hair cell to afferent synapse uses glutamate as the principal neurotransmitter. At the postsynaptic cell glutamate interacts with several subtypes of excitatory amino acid (EAA)-receptors including N-methyl-D-aspartic acid (NMDA), α-amino-3-hydroxyl-5-methyl-4-isoxazole-propionic acid (AMPA), kainic acid (KA) and metabotropic receptors. The efferent neurons are primarily cholinergic, and ACh released from afferent neurons interacts with muscarinic (mACh) and nicotinic (nACh) receptors. The efferent neurons also release calcitonin gene related peptide (CGRP), substance-P and enkephalins, which act on specific receptors (in the case of the enkephalins κ−opioid receptor in the hair cells and µ−opioid receptors  in the afferent neurons). Both the hair cells and the afferent neurons expressed the nitric oxide synthase (NOS) and produced nitric oxide (NO). Hair cells also express H_1_ histamine receptors and the afferent neurons H_3_ histamine receptors. The hair cells typically express purinergic receptors (ATP) in their apical portion.

In the inner ear, hair cells of the vestibular neuroepithelium establish synapses with afferent neurons and receive innervation from the efferent neuronal system. The main neurotransmitter of hair cell afferent synapses is glutamate; efferent neuronal synapses release acetylcholine as the primary neurotransmitter (Fig. **1**). Glutamate released at hair cell afferent synapses interacts with several subtypes of excitatory amino acid (EAA) receptor, including N-methyl-D-aspartic acid (NMDA), α-amino-3-hydroxyl-5-methyl-4-isoxazole-propionic acid (AMPA), kainic acid (KA) receptors, and metabotropic receptors [7, 38, 52, 73, 116, 137, 139]. The NMDA receptors participate in determining the basal discharge and tonic response to sustained stimuli, whereas non-NMDA receptors seem to mediate responses to high-frequency mechanical stimulation [39, 139].

Hair cells and efferent neurons also release diverse neuroactive substances, including calcitonin gene related peptide (CGRP), substance P, opioid peptides, endocannabinoids, γ-aminobutyric acid (GABA), ATP, adenosine and histamine [38, 52]. Apart from the neurotransmitters that participate in the processing of sensory information, the vestibule also receives sympathetic and parasympathetic innervation, a fact that probably accounts for the presence of catecholamines in the inner ear [47].

Vestibular system primary afferent neurons make synapses with neurons of the vestibular nuclei, where they release glutamate and, probably, aspartate as neurotransmitters. The neurons of the vestibular nuclei send glutamatergic, cholinergic and GABAergic projections to numerous parts of the central nervous system (CNS), including the cerebellum, oculomotor nuclei, contralateral vestibular nuclei, autonomic nuclei, spinal cord, thalamus and cerebral cortex [13, 60, 161]. The vestibular nuclei receive projections originating from the visual system, particularly from the accessory optical system (a system that play a role to differentiate a subject’s own movements from environmental ones) and the visual cortex. Vestibular nuclei also receive proprioceptive signals, especially from cervical regions, from the cerebellum and from diverse fibers that originate from diencephalic nuclei. The numerous and diverse synaptic inputs to the vestibular nuclei form part of the very complex integrative processes involved in postural control and gaze stabilization [131].

**Fig. (2):**
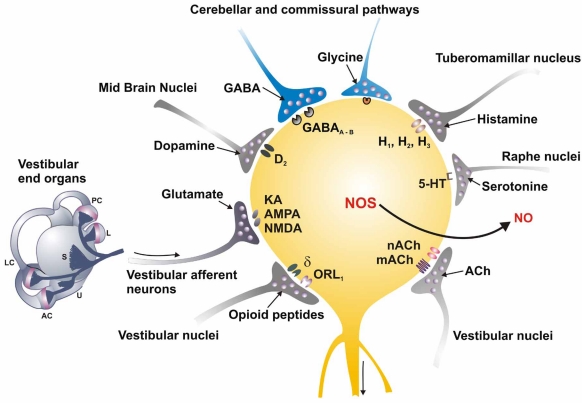
The scheme depicts the complexity of synaptic input impinging on the vestibular nucleus neurons. It is necessary to consider that the neurons of the nuclei are heterogeneous and not all cells necessarily receive all types of synaptic influences. The main synaptic input to the vestibular nuclei neurons is from the primary afferents, mediated by glutamate that interacts with NMDA, AMPA/KA and metabotropic receptors. Vestibular nuclei also receive glutamatergic synapses originating from spinal cord neurons. GABAergic fibers originating primarily from the cerebellum and from the contralateral vestibular nuclei impinge on the vestibular nucleus neurons, activating GABA-A and GABA-B receptors. Histaminergic fibers originating from the tuberomammillar nucleus act on H_1_, H_2_ and H_3_ receptors. Serotonergic fibers originating from the raphe nuclei activate 5-HT1 and 5-HT2 receptors. Intrinsic and commissural connections give rise to glycinergic fibers acting on glycine inhibitory receptors. Noradrenergic fibers originating from the *locus coeruleus* act primarily on α2 receptors, but also on α1 and β receptors. Internuclear enkephalinergic fibers that release orphanin-nociceptin F/Q acting on ORL1 receptors (*opioid-like orphan receptor*) and δ-opioid receptors also make a synaptic input to certain neurons in the nuclei. The endocannabinergic type CB1 receptors have also been detected in the vestibular nuclei. The output of the vestibular nuclei neurons is primarily by glutamatergic and cholinergic projections, but GABAergic and glycinergic projections have been demonstrated also. Finally, the vestibular nucleus neurons express the NOS and may produce NO as a cellular messenger.

The neurons of the vestibular nuclei also express NMDA and non-NMDA EAA receptors [26, 49, 158]. Among other functions, these receptors mediate the main excitatory input to the vestibular nuclei neurons that originates from the primary afferents [31, 115, 148]. EAA receptors alter their expression in a dynamic manner; for example, NMDA receptors mediate a long-lasting depression that forms a significant part of the mechanism that suppresses the activity of neurons from the vestibular nuclei following labyrinth ablation [60, 130]. In addition to the EAA receptors mediating the input from the vestibular periphery, neurons of the vestibular nuclei express a variety of neurotransmitter receptors that mediate the many integrative influences that these nuclei receive (Fig. **2**). Among these are GABA-A and GABA-B receptors and glycine receptors that show an extensive colocalization with GABA receptors, histamine H_1_, H_2_ and H_3_ receptors [15], serotonine 5HT_1_ and 5HT_2_ receptors [56], adrenergic α2 receptors, but also α1 and β receptors [124], cholinergic muscarinic (mACh) and nicotinic (nACh) receptors in all the vestibular nuclei [92], opioid receptors [146, 147], canabinoid CB1 receptors [132], neurotrophin Trk A, B and C receptors (for a review, see [27]) and finally, glucocorticoid receptors.

All these synaptic inputs to the vestibular nuclei participate in the development of various physiological processes and its study have contributed to the knowledge of the integrative physiology of the vestibular nuclei and to the many pathophysiological mechanisms involved in vestibular disorders. Commissural GABAergic inhibition seems to be exclusively mediated by GABA-A receptors, the activity of which is antagonized by bicuculline and enhanced by benzodiazepines [41, 165]. Serotonergic and dopaminergic innervation of the vestibular nuclei may account for the association that has been observed between vestibular alterations, anxiety disorders and migraine [10, 40]. The activation of glucocorticoid receptors seems to play an important role in processes leading to vestibular compensation; this would explain the beneficial action of neuroactive steroids, such as dexamethasone, in vestibular plasticity [22].

The main output of the vestibular nucleus neurons is cholinergic, but these neurons also produce other neuroactive substances such as nitric oxide, which seems to play a significant role in vestibular compensation processes [133]. Processes related with nitric oxide may account for some of the collateral effects of drugs widely used in the clinic, such as sildenafil, that can typically produce alterations in vision, including diplopia and dizziness in 2% of patients. These events have been exclusively attributed to the cardiovascular effects of the drugs, but it seems equally likely that they could be caused by alterations in vestibular activity, which, to our knowledge, has not been studied in such patients. In fact, there are reports of patients who develop severe vestibular symptoms after a single 50 mg dose of sildenafil [57].

The complexity of the synaptic connections determining the activity of the afferent neurons in the vestibular end organs, together with the intricate synaptology of the vestibular nucleus, determines that many drugs acting on synaptic receptors may influence the activity of the vestibular system. For a synopsis of the drugs acting on neurotransmitter receptors treated in this work see Table **1**.

### Drugs Acting on Excitatory Amino Acid Receptors (EAA Receptors)

Diverse pathophysiogenic models have demonstrated the role of EAA receptors in excitotoxicity [30]. Changes in intracellular ion concentrations, particularly alterations in the homeostasis of intracellular calcium secondary to EAA receptor over-activation, seem to induce oxidative stress and the generation of free radicals [128]. For this reason, it has been proposed that EAA receptor antagonists, as well as calcium channel antagonists, exert a neuroprotective action in cases of ischemia or over-activation of EAA mediated synapses. In the cochlea such over-activation may occur as a result of intense sound exposure; in the case of the vestibule, it could be caused by continuous movement. It also seems possible that an excitotoxic process may develop in postural vertigo in which semicircular canal stimulation is sustained.

Trimetazidine (1-(2,3,4 trimethoxybenzyl) piperazine hydrochloride) is an anti-ischemic agent that has been used in cardiac ischemic disturbances and tested in the symptomatic treatment of vertigo. Although its mechanism of action is not still completely defined, trimetazidine inhibits excitotoxic damage induced by the activation of EAA receptors [46], possibly by acting as a modulator of aerobic glycolysis, with a consequent decrease in lactic acid production. Trimetazidine selectively suppresses mitochondrial 3-ketoacyl coenzyme A thiolase, thereby reducing free fatty acid metabolism and balancing cardiac metabolism; these actions contribute to its anti-oxidative stress activity and to its ability to diminish mitochondrial damage in cases of ischemia [29]. Using voltage clamp recording in rat vestibular ganglion neurons, trimetazidine has been shown to block intracellular calcium increase and ionic current produced by the application of EAA receptor agonists KA and AMPA [28]. Antagonism of AMPA/KA receptors of afferent neurons can explain the beneficial effects of trimetazidine. It is possible that, in additional to its peripheral neuroprotective effect, trimetazidine could also modulate glutamatergic neurotransmission and cellular metabolism in vestibular nuclei, although no reports of this effect were found.

In clinical tests, trimetazidine is as effective as betahistine in the treatment of vertigo and dizziness [91]; multicenter studies have confirmed the utility of trimetazidine in diverse disorders that produce balance alterations [3] and, although discreet, in the reduction of the frequency and duration of vertigo in Ménière's disease patients [111]. It is worth noting that trimetazidine can induce parkinsonism, gait disorders and tremor, and at least one case of coreiform manifestations related to its use has been described [89, 129]; for this reason, its use is not advisable in patients with any predisposition to Parkinson’s or any type of tremor-related diseases.

Memantine (1-amino-3,5 dimethyiladamantane) is a low-affinity noncompetitive NMDA receptor antagonist, although some reports indicate that it also acts on serotonergic 5HT_3_ receptors, dopaminergic D_2_ receptors and cholinergic muscarinic and nicotinic receptors. Memantine has been used in the treatment of Parkinson’s disease and some dementias, including Alzheimer’s disease. In the inner ear, memantine blocks the response of the vestibular afferent neurons to NMDA receptor agonists [103], although currents through α9/α10 nicotinic receptors in cochlear outer hair cells were also blocked by memantine, with an IC_50_ of about 1 µM [104]. It has been suggested that transtympanic application of memantine might have a clinical use because it limits potential systemic effects [128]. Various case reports have proven the beneficial effect of memantine on acquired pendular nystagmus. Nevertheless, no controlled studies that demonstrate its utility in the treatment of vestibular disorders have been published [145]. Somewhat similar results have been reported for caroverine, a derivative of quinoxaline that acts like a competitive antagonist of the AMPA receptor; no controlled studies that support its use in the clinical treatment of vestibular disorders have been published.

Three noncompetitive antagonists of the NMDA receptor, including dizocilpine (dibenzo-cycle-penteno-imine, also referred to as MK-801), ketamine and phencyclidine, have a significant use in basic research and have also been the subject of clinical studies. Ketamine was used as a dissociative anesthetic. Dizocilpine was used as a neuroprotective agent in cases in which aminoglycoside use was required, under the hypothesis that cellular damage caused by aminoglycosides is due to overactivation of NMDA receptors and a subsequent excitotoxic process [14, 37, 58]. However, clinical trials showed insignificant protective action of dizocilpine against aminoglycoside ototoxicity. From our point of view, this was an expected result because there are no data indicating that hair cells, which constitute the primary cell type affected by the aminoglycosides, express NMDA receptors. In fact, streptomycin exerts an antagonistic action on EAA receptors [109] and also blocks mechanoelectrical transduction in vestibular hair cells [78]. Phencyclidine is a well known noncompetitive NMDA receptor antagonist because it is frequently used as a recreational drug (PCP or “angel dust”); it has important effects on vestibular activity. Specifically, acute poisoning with phencyclidine induced intense horizontal and vertical nystagmus due to its inhibitory action on synaptic input to neurons of the vestibular nuclei [100]. At the moment, phencyclidine has no known clinical use in vestibular pharmacology.

A more recent addition to the set of EAA receptor-acting drugs is neramexane, a novel amino-alkyl-cyclohexane derivative that is a noncompetitive NMDA receptor antagonist. This compound displaced [(3)H]-MK-801 binding to rat cortical membranes with K(i) values between 1 and 100 µM and antagonized inward current responses of cultured hippocampal neurons to NMDA in a strongly voltage-dependent manner with rapid blocking/unblocking kinetics [45]. Studies on recombinant rat α9α10 nicotinic acetylcholine receptors expressed in *Xenopus laevis* oocytes show that neramexane, as well as memantine, blocks acetylcholine-evoked responses in a noncompetitive manner [113]. The authors consider that “clinically relevant concentrations of neramexane blocked native α9α10-containing nicotinic acetylcholine receptors of rat inner hair cells, thus demonstrating a possible *in vivo* relevance in potentially unexplored therapeutic areas” [113].

### Drugs Acting on Acetylcholine Receptors

Vestibular type II hair cells and vestibular afferent neurons are innervated by efferent neurons (Fig. **1**), which exert central control of the vestibular responses based on the movement programs of the subject. The efferent neurons originate bilaterally in the brain stem, in a region located dorsolateral to the genus of the facial nerve in the proximity of the abducens nucleus and ventral to the medial vestibular nucleus [20, 108]. The efferent neurons that innervate the vestibular neuroepithelia are positive for choline acetyltransferase, acetylcholinesterase (AChE), CGRP, and enkephalins [108, 121].

The stimulation of efferent fibers produces complex effects on the activity of vestibular afferent neurons, increasing, inhibiting or having mixed biphasic effects on the electrical discharge of the afferent neurons [16, 50, 59, 154]. Studies using cloning techniques and the reverse transcriptase-polymerase chain reaction (RT-PCR) have shown that ACh nicotinic receptors in the vestibular neuroepithelia are formed essentially of α9/α10 subunits [36], although in adult rats, transcripts encoding the α2-7 and β2-4 nAChR subunits were found in the vestibular ganglia and vestibular end-organs, while α3, α5-7, α9 and β2-4 nAChR subunits were expressed in the vestibular neuroepithelia [5]. In situ hybridization histochemistry data suggest that nAChRs composed of α4 and β2 subunits are localized on afferent chalices innervating type I vestibular hair cells and that the direct cholinergic efferent innervation of type II vestibular hair cells utilizes nAChR composed of other subunits [159]. In addition to nicotinic receptors, muscarinic receptors (m1, m2 and m5) were expressed in human vestibular afferent neurons, whereas in the rat, five subtypes of muscarinic receptors (m1 - m5) are found [160]. Muscarinic receptors have been the target of many of the drugs used in the treatment of vestibular disorders.

Cholinergic input has been identified in all vestibular nuclei [92]. The application of acetylcholine to the vestibular nuclei of cats produced an activation similar to that induced by stimulation of the primary afferent neurons; the activation was enhanced by AChE inhibitors and reduced by mACh antagonists such as scopolamine. The neurons of the vestibular nuclei release ACh as a neurotransmitter; for this reason, they also express choline acetyltransferase [13, 149].

Among the drugs that modulate cholinergic activity, scopolamine and atropine have the most significant clinical application for the treatment of vestibular disorders. Other cholinergic drugs like physostigmine (also known as eserine) and neostigmine have been used experimentally to induce a motion sickness-like syndrome.

Scopolamine (also known as butylscopolamine and hyoscine butylbromide) and atropine are alkaloids of natural origin; both are nonselective competitive inhibitors of mACh receptors. Physostigmine (eserine) and neostigmine are cholinomimetics that inhibit acetylcholinesterase in the CNS and the peripheral nervous system, respectively. Both inhibitors bind to the active site of the enzyme and reduce hydrolysis of acetylcholine for up to four hours.

Scopolamine is most commonly used drug in vestibular disorders. It is one of the most effective drugs for the treatment of motion sickness; nevertheless, it has not yet been determined whether its effect takes place at the peripheral or central vestibular system [67, 160]. A recent study analyzed the literature (14 works, 1025 subjects) comparing the effectiveness of scopolamine and other agents employed in the prevention and treatment of motion sickness. The results indicate that scopolamine produced a significantly greater favorable effect than placebo and that there is no significant difference in the effectiveness of scopolamine and that of calcium channel antagonists, antihistaminics, methylscopolamine or a combination of scopolamine and ephedrine [141]. Frequent collateral effects of the use of scopolamine are blurred vision and dry mouth; occasionally, confusion also appears. Low doses of scopolamine or atropine produce a transitory tachycardia associated with the peak of their effect (90 minutes after oral scopolamine). Due to the short average life of scopolamine in plasma and its adverse effects, the clinical use of orally administered scopolamine is limited. To diminish the incidence of adverse effects, a system of transdermal dosage by means of patches has been developed. These patches are available by prescription and contain 1.5 mg scopolamine, of which 5 µg/h is released, maintaining scopolamine plasma levels approximately constant over 8-9 days [101, 119].

Contrary to the action of mACh antagonists, the administration of physostigmine, an AChE inhibitor, produced a motion sickness-like syndrome, including nausea, vomiting, discomfort, anxiety, and increases in ACTH, beta-endorphin, cortical and prolactin plasma levels [69]. In fact, physostigmine administration can be used as a model for the study of motion sickness and of the space adaptation syndrome and to detect individuals’ susceptibility to the development of these syndromes. Whereas scopolamine facilitates adaptation to movement in experimental animals, physostigmine suppresses this adaptation; interestingly, neostigmine (an AChE inhibitor that typically acts on the peripheral nervous system because it cannot cross the blood-brain barrier) does not have any significant vestibular effects. This indicates that central cholinergic systems play an essential role in the process of adaptation to movement [99].

Diphenidol (1,1-diphenyl-1-piperidinebutanol) has been used for many years in the prevention and symptomatic treatment of peripheral (labyrinthine) vertigo and associated nausea and vomiting that occur in such conditions as Ménière's disease and surgery of the middle and inner ear, as well as for the control of nausea and vomiting associated with postoperative states, malignant neoplasm, labyrinthine disturbances, antineoplastic agent therapy, radiation sickness, and infectious diseases. In patients with Ménière's disease, double-blind studies showed that use of diphenidol results in better control of symptoms than the administration of placebo [42]. Although the mechanism of action of diphenidol on the vestibular system has not yet been elucidated, it exerts an anticholinergic effect due to interactions with mACh receptors, particularly m1, m2, m3 and m4 [155]. Hence, its actions may take place at the vestibular nuclei, where a significant excitatory input is mediated by ACh receptors, and also at the vestibular periphery where mACh receptors are expressed at efferent synapses [110]. Recently, a series of selective mACh-receptor antagonists based on the diphenidol molecule has been synthesized, but they have not yet been the subject of clinical trials [155].

### Drugs Acting on Histamine Receptors

Four histaminergic receptors, designated H_1_ to H_4_, have been identified. All belong to the superfamily of G-protein coupled, seven transmembrane segment receptors. Typically, in the CNS, H_1_ and H_2_ receptors are found at postsynaptic terminals, while H_3_ receptors show a presynaptic localization in nonhistaminergic cells, where they function like heteroreceptors. H_4_ receptors are typically found outside the CNS [61, 84].

The histaminergic fibers in the CNS originate from the tuberomammillary nucleus of the posterior hypothalamus. These neurons have a spontaneous, slow, and regular action potential discharge. The activity of the tuberomammillar neurons varies in the day-night cycle: it is high during wakefulness, reduced during slow-wave sleep, and stops during rapid eye movement sleep [21]. The histaminergic neurons constitute a highly divergent system that project diffusely to various areas of the brain through ascending and descending paths [106]. Immunohistochemical studies reveal a moderately dense histaminergic innervation projecting to the four vestibular nuclei [60, 151, 168]. These fibers consist of medium diameter nonmyelinated axons that exert neuromodulatory actions in the vestibular nuclei [80].

The possibility that histamine could act like a neurotransmitter or a neuromodulator at peripheral vestibular end organs was suggested by the finding that astemizole, an H_1_-receptor antagonist that does not penetrate the hematoencephalic barrier, suppresses nystagmus in patients with chronic dizziness [68]. Later, it was demonstrated that histamine and other imidazole-containing substances increase the ampullar nerve afferent firing rate, while both H_1_ and H_2_ histamine antagonists inhibited ampullar nerve activity. A specific inhibitor of histidine decarboxylase, the enzyme that catalyzes the synthesis of histamine, reduced ampullar nerve firing in a dose-dependent manner [65]. The use of agonists and antagonists of H_1_, H_2_ and H_3_ receptors allowed investigators to determine that H_1_ and probably also H_3_ receptors are functionally expressed in vestibular end organs [53]. In the hair cells of the crista ampullaris of the guinea pig, intracellular calcium changes produced by the application of 10 µM histamine were blocked by H_1_, H_2_ and H_3_ antagonists (promethazine, cimetidine and thioperamide) [152].

Studies using RT-PCR and immunoblotting and immunocytochemical experiments in frog and mouse semicircular canal sensory epithelia showed that both frog and mouse vestibular epithelia express H_1_ receptors, although no clear evidence for H_3_ receptor expression in hair cells was found [19]. However, RT-PCR analysis showed the presence of H_3_ receptor mRNA in mouse vestibular ganglia, and H_3_ protein expression was found in vestibular neurons characterized by large and round soma, which showed positive staining for calretinin and calbindin [153].

One of the remaining problems with respect to the role of histamine in the vestibular system end organs is the origin of histamine. Histamine receptors are expressed in the inner ear, but we do not know the source of the endogenous ligands for these receptors. The possibility has been suggested that the endogenous mediator is not histamine but a related substance such as carnosine or N-acetylhistidine and that the effects of histamine antagonists occur through competition with these endogenous analogs of histamine [33, 65]. This could account for the tonic activation of histamine receptors and for the effect of histamine antagonists on the basal discharge of vestibular afferent neurons [25].

In the central nervous system, histamine modulates the activity of the second order vestibular neurons, and different effects have been observed depending on the experimental paradigm used. *In vivo* studies using iontophoresis of histamine showed a decrease in the discharge frequency of vestibular nucleus neurons in cats [76]; in contrast, *in vitro* studies using slices of the vestibular nucleus found that histamine produced an increase in the discharge frequency of medial vestibular nucleus (MVN) neurons in rats and guinea pigs [80]. Studies using whole-cell patch recording demonstrated that histamine excited lateral vestibular nucleus (LVN) neurons by activation of postsynaptic H_2_ receptors [168]. The MVN is mainly involved in both eye and head control, particularly in the horizontal plane, whereas LVN seems mainly to integrate the information of linear acceleration and gravity’s changes of the body to control vestibulo-spinal reflexes and posture [168].

Among the antihistaminics, betahistine, diphenhydramine (usually in combination with theophylline), meclizine, its derivate cyclizine, and promethazine have been the most commonly used in the medical treatment of vertigo. In Europe, betahistine is more frequently used. A prospective study in England demonstrates that, for the treatment of Ménière's disease, 92% of doctors prescribe betahistine [134]. In contrast, in the United States, the antihistaminics most commonly used in the treatment of vestibular disorders are diphenhydramine, meclizine (1[(4-chlorophenyl)-phenylmethyl]-4-[(3-methylbenzyl)methyl] piperazine), its derivate cyclizine and promethazine. The phenothiazines (promethazine and prochlorperazine) are the most frequently prescribed antiemetic drugs [96]. All these antihistaminics are primarily H_1_ receptor antagonists, but they also have an anticholinergic effect that is more remarkable in the case of promethazine [166]. Their actions are both central and peripheral, although the central action seems essential because antihistaminics that do not cross the blood-brain barrier did not show usefulness in the treatment of vertigo. All of them cause an important sedation and, due to its potential anticholinergic effect, meclizine must be administered with caution in patients with asthma, glaucoma or prostate hypertrophy.

Although betahistine is not approved for use in the management of vestibular syndromes in the United States, it is the drug most commonly used in the European countries, Canada and Latin America for the treatment of Ménière's disease [134]. Controlled studies demonstrate that betahistine was the most effective when compared with cinnarizine, clonazepam, flunarizine or Ginkgo biloba administered for 120 days to patients diagnosed with Ménière's disease. For patients with peripheral vestibular alterations different from Ménière's disease, betahistine was as effective as cinnarizine or clonazepam, and all three were more effective than flunarizine or EGb 761 (standardized extract of Ginkgo biloba) [43]. Studies using a combination of cinnarizine and dimenhydrinate indicate that this combination could be more effective than betahistine in the treatment of patients with vertiginous syndromes of peripheral origin, making it a good and safe alternative to standard therapy with betahistine for the treatment of Ménière's disease acute episodes and in long-term therapy [54, 102].

Betahistine is an analog of histamine that has an antagonistic action on H_3_ receptors [9]. It also has a partial, weak agonist effect on H_1_ and H_2_ receptors [61, 162]. In rat brain stem slices, betahistine produced a slight excitatory action on neurons of the MVN and, in spite of its apparent weak potency, significantly reduced the excitatory effect of histamine [64]. When administered in a sustained form to cats, betahistine significantly alters histamine turnover in neurons of the tuberomammillary nucleus [150]. In the vestibular end organs of rodents and amphibians, betahistine, as well as its metabolite, 2-(2-aminoethyl) pyridine, diminishes the discharge frequency of the vestibular afferent neurons [17, 18, 25].

Since the introduction of betahistine, it has been reported that its administration significantly reduces the incidence and severity of vertigo and produces a decrease in the incidence of nausea and emesis [1, 51, 75, 79, 81, 97, 98]. Its clinical effect has been attributed to increased blood flow in the vestibular system and cochlea [90]. Electrophysiological studies have established that the clinical action of betahistine is most probably produced by an inhibitory influence exerted at the vestibular nuclei and at the peripheral end organs; this inhibitory action modulates the afferent sensory input and the release of other neurotransmitters and reestablishes the bilateral balance of activity in the afferent neurons [25, 53, 138].

It is worth noting that antihistaminics cause a depression of the central nervous system, which imposes a serious limitation when the activities of the subject require alertness; for this reason, in certain cases, it is possible to use antihistamines in combination with amphetamines [107]. Promethazine is especially known to cause drowsiness, which is often counteracted by ephedrine in a combination known as “the Coast Guard cocktail.”

### Drugs with Effects on GABA Receptors 

The possible role of GABA in the vestibular periphery has been widely discussed without reaching a clear definition of its function in synaptic transmission in vestibular end organs [62, 94, 157]. GABAergic pathways from the cerebellum and commissural fibers from the contralateral nuclei exert a powerful inhibitory input on the vestibular nuclei, activating both GABA-A (ionotropic) and GABA-B (metabotropic) receptors [60, 131].

Diazepam is the most widely used benzodiazepine in the treatment of vestibular disorders, although lorazepam and clonazepam are also frequently used. Clonazepam is particularly useful in the treatment of migraine-related vertigo and postural vertigo. Diazepam, a typical GABA-A receptor agonist, significantly reduces the spontaneous electrical activity of neurons in the medial vestibular nuclei, exerting both pre- and post-synaptic actions on diverse groups of neurons [62, 63, 125]. The inhibitory effect of the benzodiazepines on the electrical activity of the vestibular nuclei leads to its therapeutic effect in vertiginous syndromes, apart from their sedative and anxiolytic action that significantly contributes to the well-being of patients [55, 166].

Baclofen, a selective agonist of the GABA-B receptor, has shown a promising effect, particularly in the treatment of uncompensated vestibular asymmetry in animal models [55]. Baclofen presumably acts by enhancing inhibition in vestibular nuclei and related networks, consequently reducing nystagmus in patients with vestibular alterations. A few studies showed that baclofen improves periodic alternating nystagmus [167]. In contrast, the GABA-A receptor agonist gabapentin (probably also acting on calcium channels) acts on pendular nystagmus [145].

### Drugs with Action on the Opioid Peptide Receptors

Like other neurotransmitters and their receptors, enkephalins and opioid receptors are expressed in both the central and the peripheral nervous systems. Opioid peptides have been detected in auditory and vestibular efferent neurons, where they colocalize with the major neurotransmitter, acetylcholine. RT-PCR, in situ hybridization, Western blots and immunohistochemistry have shown the expression of Mu-opioid receptor (MOR) in Scarpa's ganglia and cristae ampullaris of the rat. MOR transcript was detected in the soma of the vestibular afferent neurons. These results indicate that MOR may mediate some of the synaptic influences of vestibular efferent neurons on afferent neurons [114]. Functionally, the opioid receptors exert a complex neuromodulatory action on vestibular end organs. They exert an inhibitory presynaptic action on hair cells and an excitatory postsynaptic action in afferent neurons. In hair cells, the activation of kappa-opioid receptors inhibits the calcium current, decreasing afferent activity. The activation of Mu-opioid receptors on afferent neurons potentiates the action of excitatory amino acids, increasing the afferent activity [156]. This type of opposed pre- and postsynaptic action is typical of opioid receptors and accounts for the state-dependent activity of opiate neurotransmitters. It is worth noting that the α9/α10 nAChR, with its peculiar sensitivity to various agents, is a target for modulation by endomorphin-1 and dynorphin-B, efferent cotransmitters in the inner ear [87].

The opioids also make an important contribution to the operation of vestibular nucleus neurons [131]. Enkephalin-containing cell bodies and nerve terminals are found in the medial vestibular nucleus, and a few substance P-containing neurons have been found in vestibular nuclei [93]. Microiontophoretic application of enkephalin produced an inhibition of the electrical discharge of neurons of the medial vestibular nucleus, as well as a decrease in its response to glutamate, suggesting that opioids modulate the afferent input to the nuclei [74]. The agonist of the opioid receptor like-1 (ORL-1) nociceptin, as well as [D-Ala2, D-Leu5]-enkephalin (DADLE: an agonist of the delta opioid receptor), also exert an inhibitory effect on the electrical activity of MVN neurons. The application of nociceptin in the rat diminishes the gain of the vestibulo-ocular reflex [146, 147].

The finding that opioids affect the excitability of the inner ear explains why diverse drugs of abuse, such as heroin and more recently dihydrocodone, can produce significant alterations in hearing and balance. In fact, there are reports of the occurrence of a Ménière's like-syndrome after the use of epidural morphine [86]. The combination of phentanyl (a synthetic primary MOR agonist 100 times more potent than morphine) and droperidol (an antidopaminergic drug used as an antiemetic and antipsychotic) has been used for the treatment of Ménière's disease crises; this mixture has a rapid action and results in a complete suppression of vestibular activity in normal subjects and in those with Ménière's disease [32]. Administration of a single dose of phentanyl and droperidol combination (droperidol 5 mg, phentanyl 0.1 mg) to patients undergoing acute episodes of vestibular disease (vestibular neuronitis and Ménière's disease) was effective in the following symptoms and/or signs: nausea, vertigo, nystagmus, the positive past-pointing test and the Romberg test [71]. Droperidol alone is also remarkably effective in depressing vestibular disturbance, regardless of etiology. However, no difference has been found between the therapeutic efficacies of intramuscular injection of droperidol and dimenhydrinate for the treatment of acute peripheral vertigo [66].

### Drugs Acting on Amine Receptors

Drugs that affect biogenic amine receptors have been widely used in the therapy of diverse vestibular disorders; there is consensus that their effects are exerted essentially at the central nervous system, not specifically in vestibular nuclei, although these have an important noradrenergic, serotonergic and dopaminergic input [60].

Amphetamines improve tolerance to stimulation with rotations [163]. The use of amphetamines in combination with scopolamine or antihistaminics increased the antivertiginous effect and reduced the secondary sedation produced by antihistaminics and anticholinergic drugs. Ephedrine seems to be as effective as amphetamines in increasing the effectiveness of drugs used for the control of movement disease, but without the addictive potential of amphetamines.

The application of dopamine (0.1-1 mM) and D1 and D2 agonists induced a dose-dependent and reversible decline in the resting discharge frequency of vestibular afferents in the isolated frog vestibule [6]. The responses of afferent neurons to EAA agonists were inhibited by D1 and D2 agonists, implicating both subtypes of dopamine receptors in the modulation of both ionotropic and metabotropic glutamate receptors. The results obtained suggest that dopamine may interact with both D1 and D2 receptor subtypes that are most likely located postsynaptically on the afferent neurons, where they may form part of the efferent control of vestibular input [6]. These findings coincide with those of previous studies that indicate the participation of catecholamines in vestibular neurotransmission [47] and contribute to understanding the mechanism of action of dopaminergic-related drugs, such as droperidol, in the treatment of vestibular disorders. In fact, sulpiride, which is a selective antagonist at postsynaptic D2 receptors, has been used as a “vestibular sedative”.

### Drugs Acting on Cannabinoid Receptors 

It has recently been established that cannabinoids play an important role in the activity of neurons of the vestibular nuclei through the activation of cannabinoid CB1 type receptors [132]. There is also evidence suggesting the expression of cannabinoid receptors in vestibular end organs [23]. These results indicate the possibility of using cannabinoid-related drugs in the treatment of vestibular alterations, particularly now that several drugs with effects on these receptors are available [120]. Although much information is available on the use of cannabinoids to control chemotherapy-induced alterations, including dizziness and emesis, as far as we have been able to determine, no double-blind and well controlled studies addressing cannabinoid use in vestibular disorders are available.

## DRUGS ACTING ON VOLTAGE-GATED IONIC CHANNELS

Almost all cells of an organism express a set of membrane ion channels. Nevertheless, ion channels acquire special relevance in the physiology of excitable cells. The definition of an important set of channelopathies that affect inner ear function and the identification of the functional roles of diverse subtypes of ion channels in the inner ear has allowed substantial expansion of the use of drugs with effects on these membrane proteins. Knowledge of ion channel function offers therapeutic possibilities for the rational design of new drugs and drug combinations. Importantly, it has been proposed that pathologies as complex as Ménière's disease might have their origin in channelopathies, particularly in those cases in which there is a clear familial expression of the disease [44]. For a synopsis of the drugs acting on voltage-gated ionic channels treated in this work see Table **2**.

### Calcium Channel Blockers

The ubiquity of calcium channels determines that calcium channel blockers can exert diverse effects at the central and at the peripheral vestibular system. From the pharmacological point of view, the participation of calcium channels in neurotransmitter release at synaptic terminals constitutes the primary therapeutic target of calcium channel blockers, although the calcium-activated potassium current may also be a significant target contributing to the action of these drugs [34]. Neurotransmitter release in hair cells is mediated by high voltage-activated calcium channels (Ca_V_1.3) that can be blocked by dihydropyridines [4, 8, 11, 112].

The expression of type L, N, P and Q calcium channels has been demonstrated in primary afferent neurons of the vestibular system [24]. All these channels, identified on the basis of their biophysical and pharmacological characteristics, participate in generation of the action potential discharge and in control of the excitability of afferent neurons by the activation of the calcium-activated potassium current [85]. The calcium currents that occur in the afferent neurons also participate in neurotransmitter release at the vestibular nuclei.

Neurons of the vestibular nuclei express high threshold L and N-type calcium channels as well as low threshold T-type calcium channels [126, 127]. Hence, the calcium channel blockers affect the input and output of information of the vestibular nuclei. Depending on the functional role of the subtypes of channel in each synapse, the effect of some blockers could be greater than others, leading to selectivity [135]. At synapses in the central nervous system, the calcium channels participating in neurotransmitter release are N or P/Q-type. These channels are not blocked by the dihydropyridines. In contrast, in the hair cell to afferent neuron synapse, they are dihydropyridine sensitive L-type channels. Since the majority of the agents used in clinics are derived from dihydropyridines the effect of these drugs is primarily peripheral.

The most commonly used calcium channel blockers for the management of vestibular disorders are nimodipine, nitrendipine (a dihydropyridine with long lasting effect) and verapamil. Other long lasting dihydropyridines such as amlodipine, felodipine, nicardipine and nifedipine are seldom used [55, 83, 123].

The piperazine derivates cinnarizine and flunarizine, both of which have an antihistaminic action, have been widely used in the treatment of vestibular disorders. Cinnarizine, besides its L-type calcium channel blocking action [8], acts as a blocker of the pressure-sensitive potassium channels. This may be relevant in the vestibule, since this last type of channel can be activated when inner ear endolymphatic hydrops develops [34, 35]. In addition, cinnarizine and flunarizine have antihistaminic action (acting on H_1_ receptor) and potential (although very weak) nicotinic receptor antagonism. Disappointingly, no studies have evaluated the exact role of these actions in the therapeutic effect of these drugs [117].

The dihydropyridines nimodipine and nitrendipine are typical L-type calcium channel blockers [95] and exert their effect on the calcium currents in hair cells, inhibiting afferent neurotransmitter release [4, 112]. Verapamil, although it belongs to a different chemical class of fenilalkilamines typically used for its antiarrhythmic action, has also been successfully used in the management of vestibular migraine syndrome [118].

There are reports that suggest a significant effectiveness of cinnarizine on the management of vertigo of peripheral origin. A multinational and multi-center double blind study compared the effectiveness of cinnarizine (150 mg per day) with that of nimodipine (30 mg three times per day). After 12 weeks of treatment, nimodipine decreased the incidence of moderate attacks of vertigo by 79%, and those of severe vertigo by 85%; whereas cinnarizine reduced attacks of moderate vertigo by 66% and of severe vertigo by 90% [105].

Gabapentin (Neurontin), which was originally thought to act exclusively on GABA receptors, also has a blocking effect on calcium channels, specifically binding to the α2δ subunit of these channels [82]. This drug has been used in the treatment of vertigo and certain types of nystagmus of central origin [142].

Independently of their effects at the vestibular level, the blockade of calcium channels has a negative inotropic effect and antimigraine action. Calcium channels also modify diverse cellular signals related to apoptosis. In fact, because patients with vestibular alterations have a high prevalence of migraine, the blocking of calcium channels (particularly by verapamil and nimodipine) offers an additional advantage because of its anti migraine effect [118].

### Drugs Acting on Sodium Channels

Diverse types of sodium channels are expressed in the inner ear neuroepithelia. Among them, the TTX-sensitive Na_V_1.7 plays a fundamental role in the action potential discharge in afferent neurons and in the neurons of vestibular nuclei. Sodium currents participate in the ontogeny of hair cells and mediate the generation of the action potential in neurons. Pharmacological modification of sodium channels produces significant changes in the excitability and discharge pattern of the afferent neurons [26, 136].

In a rat experimental model, transtympanic infusion of lidocaine produces a vigorous spontaneous nystagmus that reaches its maximum after 20 min of drug administration [88]. This is consistent with the known sodium channel blocking action of lidocaine. Its use produces a functional, reversible ablation of the inner ear. There are clinical reports indicating that transtympanic infusion of lidocaine with dexamethasone could have a significant beneficial effect in the management of acute vertigo and in the long-term management of patients with Ménière's disease. With the use of transtympanic lidocaine in a group of 113 patients, 83% experienced an immediate lightening of the sensation of fullness of the ear and dizziness; a year after treatment 69% of these patients still showed remission of symptoms [122]. Nevertheless, in these studies lidocaine was combined with corticoids; therefore, it is difficult to judge which drug caused the effect. More recently, the use of transtympanic lidocaine in treatment of Ménière's disease has been reevaluated. Transtympanic instillation of a solution of 4% lidocaine with furfuryladenine produced a remarkable improvement in the vestibular symptoms in 87.5% of patients; 67% of these patients were free of attacks after approximately 26 months of follow up. In this work, as in the previously cited studies, lidocaine was coapplied with other drugs, in this case N(6)-furfuryladenine, which is a natural stable secondary DNA damage product with very well defined cytokine and anti-aging properties that has been linked to oxidative processes in cells [2, 12].

Carbamazepine, an anticonvulsant structurally similar to tricyclic antidepressants, is used in the management of epilepsy, bipolar disorders and trigeminal neuralgia. Its action is related to the stabilization of the inactivated state of the voltage-dependent sodium channel; consequently, carbamazepine reduces cellular excitability. Carbamazepine has been used in the treatment of tinnitus. In studies of tinnitus induced by salicylates in rat models, 15 mg/kg i.p. carbamazepine (but peculiarly, not 5 mg/kg or 30 mg/kg) suppressed the behavioral manifestations of tinnitus [169]. Vertiginous alterations such as vestibular paroxysms have been treated with low doses of carbamazepine (200-600 mg per day) or oxocarbamazepine; these agents show a rapid beneficial effect. Carbamazepine must be considered a therapeutic alternative to the use of gabapentine, valproic acid or phenytoin [145]. Carbamazepine and oxocarbazepine have also been used in the treatment of vertigo and particularly in paroxystic disorders. In general, agents used for the treatment of epilepsy seem promising in the treatment of vertigo of different origins [55, 72, 77].

### Drugs Acting on Potassium Channels

Potassium channels are widely expressed in the nervous system. Among ion channels, they exhibit the greatest diversity; at least three major families of potassium channels have been defined (2-, 4- and 6-transmembrane segment groups, each with a significant number of subfamilies). Potassium channels play a significant role in many channelopathies, and many drugs act on these channels. However, in the management of vestibular disorders, only one drug, 3,4-diaminopyridine, a blocker of voltage-activated K^+^ channels (Kv1 family with six transmembrane segments in the alpha subunit), has been used successfully for the management of downbeat nystagmus [143]. In controlled studies against placebo, it was demonstrated that 3,4-diaminopyridine produced a significant control of the nystagmus that appears in patients with type 2 (EA2) episodic ataxia [144]. The rationale behind the study was that potassium channel blockers presumably increase the excitability of cerebellar Purkinje cells, thereby augmenting the inhibitory influence of these cells on vestibular and cerebellar nuclei [48, 70]. On the basis of these results, it has been proposed that aminopyridines could have abundant utility in diverse oculomotor disorders and in disorders of cerebellar origin [145].

Our research group has generated evidence that low threshold voltage-activated potassium current has a significant role in the control of the activity of vestibular afferent neurons [110, 140]. This current is modulated by the activation of mACh receptors (thus, it is called the M-current). Aside from the fact that the KCNQ family of potassium channels are also expressed in transport epithelia involved in potassium movement to the endolymph, the functional role of the M-current in vestibular afferent neurons allows us to suggest the use of retigabine, an activator of the M-current, and of linopirdine, a blocker of the M-current, as drugs with significant potential for the management of vestibular disorders that may result from an imbalance of the afferent neuron activity incoming from each vestibule.

## CONCLUSION

Although a large pharmacological arsenal for the treatment of vestibular disorders exists, due to the complexity of this organ and to the adaptive processes that compensate for vestibular deficits thereby producing a complex evolution of vestibular symptomatology, it is extremely difficult to evaluate the effectiveness of commonly used drugs in the treatment of vestibular disorders. Particularly in the case of Ménière's disease, the natural evolution of the disease tends to limit the symptoms. In some cases, only the recurrence of vertigo episodes, or the evolution of auditory loss, allows the clinician to evaluate the real utility of the pharmacological treatment. Critical analysis of the existing literature reveals that there is a significant lack of information defining the real utility of the diverse drugs used in the clinical treatment of vestibular disorders and that there is a lack of well-controlled clinical studies utilizing comparative analysis with high levels of reliability. However, the development of basic studies addressing drug actions at the molecular, cellular and systems level, combined with reliable and well controlled clinical trials, will provide the scientific basis for new rational strategies for the treatment of vestibular disorders.

## Figures and Tables

**Table 1:** Drugs with Effect on Neurotransmitter Receptors

Trimetazidine	Noncompetitive EAA-R antagonist	Ischemia or conditions cursing with over-activation of the afferent input
Memantine	Noncompetitive NMDA-R antagonist. With serotonergic 5HT_3_-R, dopaminergic D_2_-R, and cholinergic mACh-R and nACh-R antagonism	Used to control acquired pendular nystagmus
Dizocilpine	NMDA-R antagonist	Neuroprotective agent in cases in which aminoglycoside use was required
Neramexane	Noncompetitive NMDA-R andα9α10-containing nACh-R antagonist	Phase III clinical trials for tinnitus control. ¿Can it be useful for ischemia or as neuroprotective for vestibular disorders?
Scopolamine and atropine	Competitive mACh-R antagonists	The most commonly used drug in vestibular disorders. Most effective drugs for the treatment of motion sickness
Diphenidol	Anticholinergic effect due to interactions with mACh-R	Prevention and symptomatic treatment of peripheral (labyrinthine) vertigo and associated nausea and vomiting. Control of nausea and vomiting of any origin
Betahistine	H_3_-R antagonistic and a partial, weak agonistic of H_1_ and H_2_ receptors	Most used drug in the Ménière's disease control in Europe. Reduces the incidence and severity of vertigo and the incidence of nausea and emesis
Diphenhydramine	Antagonist of H_1_ and of ACh receptors	Most used drug in the Ménière's disease control in USA
Meclizine and cyclizine	Antagonist of H_1_ and of ACh receptors	Commonly used in combination with betahistine or diphenhydramine in the Ménière's disease control
Promethazine	Antagonist of H_1_ and of ACh receptors	Treatment and prevention of vertigo
Diazepam, lorazepam and clonazepam	GABA-A receptor antagonists	Therapeutic effect in vertiginous syndromes, apart from their sedative and anxiolytic action that significantly contributes to the well-being of patients
Baclofen	GABA-B receptor antagonist	Used to control periodic alternating nystagmus
Gabapentin	GABA-A receptor agonist Slight Ca^2+^ channel block	Used to control pendular nystagmus
Phentanyl	Synthetic primary MOR agonist	Used in the treatment of Ménière's disease crises and vestibular neuronitis in combination with droperidol
Droperidol	Dopaminergic receptor antagonist	Usually an antipsychotic. Useful for treatment of acute peripheral vertigo
Sulpiride	Selective D_2_ receptor antagonist	Used as a vestibular sedative

Excitatory amino acid receptor, EAA-R; N-methyl-D-aspartic acid receptor, NMDA-R; Type 3 serotonergic receptor, 5HT_3_-R; Type 2 dopamine receptor, D_2_-R; Nicotinc acetylcholine receptor, nACh-R; Muscarinic acetylcholine receptor, mACh-R; Type 1, 2 or 3 histamine receptor, H_1_-, H_2_- or H_3_; Type A or B γ-aminobutyric acid, GABA; µ-opioid receptor, MOR.

**Table 2:** Drugs with Effect on Voltage-Gated Ionic Channels

Dihydropyridines: nimodipine, nitrendipine	L type Ca^2+^ channel blocker	Management of vertigo of peripheral origin
Cinnarizine and flunarizine	L type Ca^2+^ channel blocker Slight H_1_ antihistaminic action. Potential antagonism of nACh-R Cinnarizine also acts as a pressure-sensitive potassium channel blocker	Management of vertigo of peripheral origin
Verapamil	L type Ca^2+^ channel blocker	Management of vertigo of peripheral origin
Lidocaine	Na^+^ channel blocker	Transtympanic infusion of lidocaine with dexamethasone for the management of acute vertigo and in the long-term management of patients with Ménière's disease
Carbamazepine	Stabilizes the inactivated state of the voltage-dependent Na^+^ channel	Treatment of vestibular paroxysms
3,4-diaminopyridine	Blocker of Kv1 (KCNA) family of voltage-activated K^+^ channels	Control of the nystagmus in patients with type 2 (EA2) episodic ataxia

Nicotinc acetylcholine receptor, nACh-R; Type 1 histamine receptor, H_1_.
